# Book Reviews

**Published:** 1799-05

**Authors:** 


### PHARMACY AND MATERIA MEDICA.

*Dictionario elemental de Farmacia,* &c.—" *An Elementary Dictionary of Pharmacy*; *or the Application of the Principles of modern Chemiſtry to the chief Operations of Pharmacy; with an extenſive new Nomenclature, and a complete Table of Contents:* By Don Manuel Hernandes de Gregorio, Apothecary to the King. 2 volumes, ſmall 4to. 300 pp. each. *Madrid.*

It is with much ſatisfaction we obſerve the attention and ſucceſs, with which the Spaniards cultivate the different branches of medical and phyſical ſcience. The preſent work is commendable not only for conciſeneſs, but likewiſe for a ſatisfactory account of the moſt important particulars relative to this department of phyſic.

The explanatory introduction is well written; the author's definition of pharmacy and its relation to medicine is accurate; his diſtinction between ſimple and compound bodies is correct, as well as his ſpecification of their different genera, and the elementary view of the three kingdoms of nature.

In the Pharmaceutical Dictionary the different articles are well arranged, and clearly defined; a proof that the author is converſant with modern diſcoveries. Under the term 'Vegetables', he treats of the philoſophy of botany, and deſcribes the uſual plants, by the diſtinction of officinal and botanical. The whole is concluded with a ſynonymical view of the old and new chemical nomenclature.

*Traité des proprietés, uſages, et effets de la Douce amère,* &c.—" *A Treatiſe on the Properties, Uſes, and Effects of the Solanum ſcandens, in the treatment of Ringworms or Tetters:* By Cit. Carrere. 8vo. (3 livres). *Paris.* Moutardier.

This work has been ſanctioned by a moſt flattering report made by C. C. Geoffry and Audrey, a committee appointed by the medical Society

Society of Paris. It is, according to French ideas of Therapeutics, a valuable acceſſion to the obſervations already publiſhed by the author, on this ſpecies of night-ſhade.

*Des Charactères, du Traitement et de la Cure des Dartres,* &c.—"*On the Nature, Treatment, and Cure of Ringworms or Tetters; Palſy of the Lower Extremities; Convulſions; the Hooping Cough; Epilepſy, Tetanus,* &c. *by the uſe of the Rhus radicans, &c.* By A. Dufresnoy. 8vo. (3 livres). *Paris.* Méquignon,

Although this publication belong more properly to the next following head than to the preſent, as its title is rather of a therapeutical than pharmaceutical complexion; yet, as the preceding work is principally written on the ſame ſubject, and as we are no advocates for the introduction of *empirical practice,* (or what tends to the ſame diſgraceful end—the encouragement of *ſpecifics*), we have here placed them together, and conſigned them both to the benefit of the dabbling apothecary, rather than to the peruſal of the reflecting practitioner.—In juſtice, however, to the author of this volume, we ſhall repeat what he has himſelf aſſerted, that after a great number of experiments made in the courſe of *ten years,* with the *rhus radicans,* and a ſpecies of the narciſſus, which he terms *narciſſe de près,* he has been able to perform a variety of cures in the diſeaſes enumerated in the title page, by preparations made of theſe two plants, which have hitherto been little known or employed as medicines.

